# A model for studying the energetics of sustained high frequency firing

**DOI:** 10.1371/journal.pone.0196508

**Published:** 2018-04-30

**Authors:** Bela Joos, Michael R. Markham, John E. Lewis, Catherine E. Morris

**Affiliations:** 1 Department of Physics, University of Ottawa, Ottawa, Ontario, Canada; 2 Center for Neural Dynamics, University of Ottawa, Ottawa, Ontario, Canada; 3 Department of Biology, The University of Oklahoma, Norman, Oklahoma, United States of America; 4 Department of Biology, University of Ottawa, Ottawa, Ontario, Canada; 5 Neuroscience, Ottawa Hospital Research Institute, Ottawa, Ontario, Canada; Georgia State University, UNITED STATES

## Abstract

Regulating membrane potential and synaptic function contributes significantly to the energetic costs of brain signaling, but the relative costs of action potentials (APs) and synaptic transmission during high-frequency firing are unknown. The continuous high-frequency (200-600Hz) electric organ discharge (EOD) of *Eigenmannia*, a weakly electric fish, underlies its electrosensing and communication. EODs reflect APs fired by the muscle-derived electrocytes of the electric organ (EO). Cholinergic synapses at the excitable posterior membranes of the elongated electrocytes control AP frequency. Based on whole-fish O_2_ consumption, ATP demand per EOD-linked AP increases exponentially with AP frequency. Continual EOD-AP generation implies first, that ion homeostatic processes reliably counteract any dissipation of posterior membrane E_Na_ and E_K_ and second that high frequency synaptic activation is reliably supported. Both of these processes require energy. To facilitate an exploration of the expected energy demands of each, we modify a previous excitability model and include synaptic currents able to drive APs at frequencies as high as 600 Hz. Synaptic stimuli are modeled as pulsatile cation conductance changes, with or without a small (sustained) background conductance. Over the full species range of EOD frequencies (200–600 Hz) we calculate frequency-dependent “Na^+^-entry budgets” for an electrocyte AP as a surrogate for required 3Na^+^/2K^+^-ATPase activity. We find that the cost per AP of maintaining constant-amplitude APs increases nonlinearly with frequency, whereas the cost per AP for synaptic input current is essentially constant. This predicts that Na^+^ channel density should correlate positively with EOD frequency, whereas AChR density should be the same across fish. Importantly, calculated costs (inferred from Na^+^-entry through Nav and ACh channels) for electrocyte APs as frequencies rise are much less than expected from published whole-fish EOD-linked O_2_ consumption. For APs at increasingly high frequencies, we suggest that EOD-related costs external to electrocytes (including packaging of synaptic transmitter) substantially exceed the direct cost of electrocyte ion homeostasis.

## Introduction

Analysis of mammalian brain energetics identifies electrical signaling as the major consumer of ATP with molecular processes underlying synaptic transmission incurring the highest costs [1–3]. For brain, the costs of low frequency electrical signalling arising from various aspects of ion homeostasis have been analyzed and summarized (see Table 2A in Ref. [3]). Changes in ion concentrations due to synaptic inputs and action potentials (APs) are reversed by the action of energy-consuming ion pumps. This direct link allows energy consumption to be estimated from Na^+^-entry [1, 4] which drives the 3Na^+^/2K^+^-ATPase activity (at 1 ATP/3 Na^+^ expelled). Under the low-firing frequency conditions (4 Hz) typical of mammalian cortex, Na^+^ (and Ca^+2^) entry through postsynaptic receptor channels is estimated to be much larger than that through AP-related voltage-dependent Na+ (Nav) channels. Synaptic processes are thus thought to dominate energetic costs in these conditions, but whether this is the case for other firing regimes is not known.

**Fig 1:**
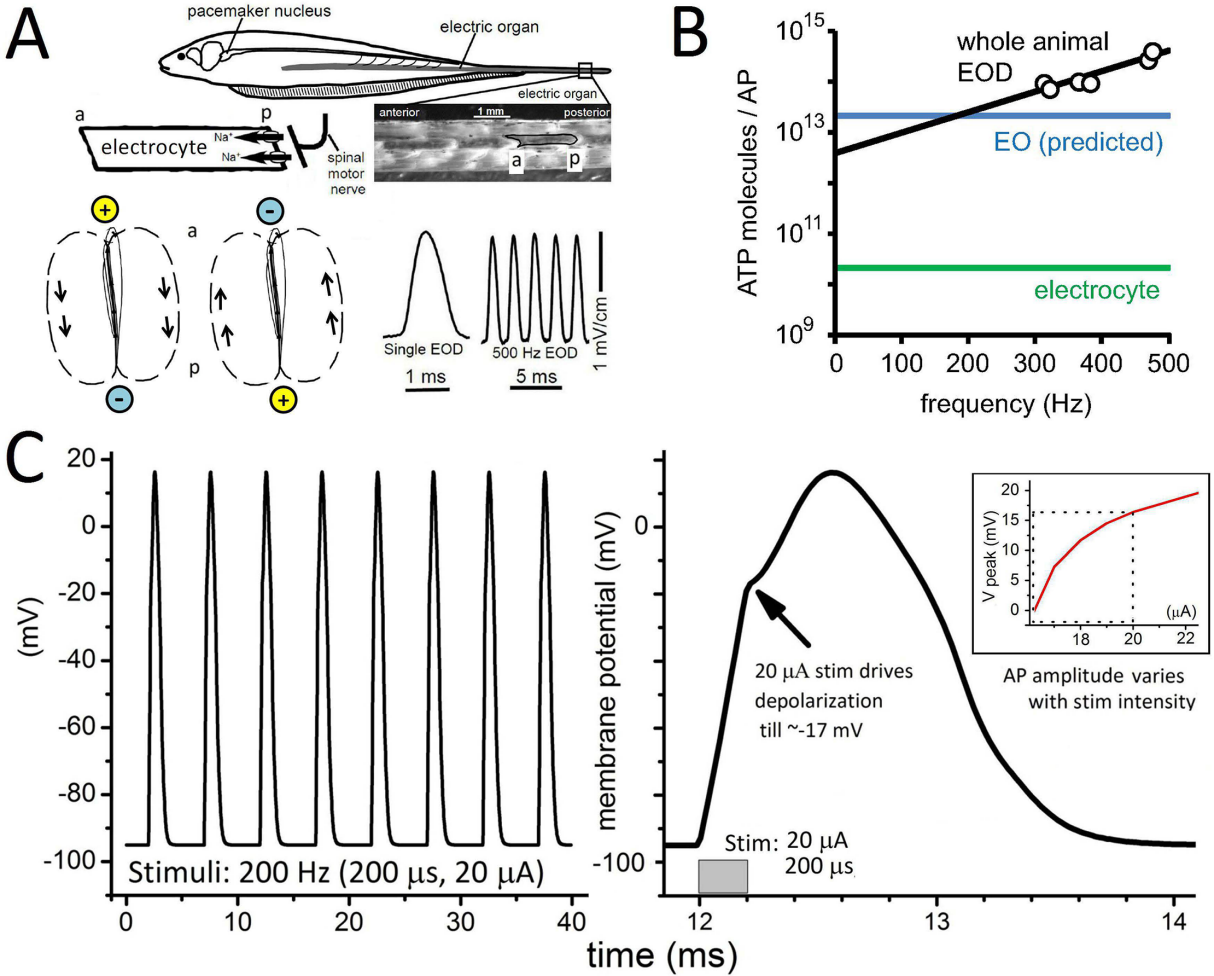
Background information. **A.** (modified from Ref. [5]) *Eigenmannia*, showing the electric organ (EO) location and the anterior(a)/posterior(p) disposition of its constituent electrocytes, each innervated posteriorly by a (central pacemaker driven) cholinergic electromotor neuron synapse (i.e., electroplaque). Due to the rapidly alternating head-positive/head-negative polarization from the continual EO discharge (EOD), the fish in its aqueous medium approximates an oscillating dipole [6]. **B.** Plot from Ref. [6], summarizing the unexpected observation (calculated from whole fish O_2_ consumption data linked to *Eigenmannia* EOD frequencies) that APs cost more as AP frequency increases. **C**. Left, an MKZ-simulated AP train (gNa_max_ = 700 μS) driven by pulsatile I_clamp_ as labeled, and right, one AP with the time scale expanded to make evident the apparent threshold region (~-17 mV) (inset: V_peak_ as a function of I_stim_).

The weakly electric fish, *Eigenmannia*, generates a high-frequency electric organ discharge (EOD) for electric sensing (**Fig 1A**). The electric organ (EO), an array of electrocytes derived from skeletal muscle (but with no contractile machinery), is a relatively simple and homogeneous excitable cell tissue. Electrocytes are posteriorly innervated syncytial cells, each ~1 mm long, with capillary beds at either end. EOD frequency is set centrally and conveyed to electrocytes via cholinergic nerve terminals at their posterior membranes. Regular and unremitting synaptic triggering of action potentials (APs) in synchronously firing electrocytes produces an externally detectable EOD at an individual-specific frequency within in the species range (200–600 Hz).

For brain energetics in general, functional magnetic resonance imaging reports on localized regions of elevated neural activity via local/temporal variations in O_2_-rich blood flow [7, 8]. However, tissue complexity and low spatial resolution impedes the study of discrete excitable cell activities, making interpretation of images problematic [9]. *Eigenmannia* is special in that whole-animal O_2_ consumption (and thence ATP usage) can be linked to a uniform unremitting excitability process, the EOD [9]. Like skeletal muscle fibers (from which electrocytes derive), the EOD producing electrocytes have cholinergic synapses and capillaries that ramify extensively with fine invaginations of the plasma membrane [5, 10, 11]. Being non-contractile, electrocytes are physiologically simpler than skeletal muscle. It seems worth exploiting this simplicity to work out how cyto- and tissue architecture interact with the molecular machinery of excitability to so reliably yield the EOD. Although the spatiotemporal variations in electric potential surrounding the intact discharging fish has been well-described [12, 13], it is not established where and for which specific processes ATP is used to maintain this oscillating discharge. Models are required to describe the underlying cellular and tissue level processes and then predict the known whole animal EOD pattern.

Monitoring EODs *in vivo* allows for precise quantitation of various aspects of *Eigenmannia*’s high-frequency excitability [12, 14]. Exploiting this, Lewis et al [6] found that the inferred ATP consumption per EOD-linked AP is not constant across all frequencies as generally assumed [1, 15], but instead increases exponentially with EOD frequency (see **Fig 1B**). This indicates that the energetics of high frequency excitability need more attention. Given the potential utility of *Eigenmannia* as a tool, a sound mathematical model for its EOD-producing machinery is required. Building on a previously-proposed model of a single electrocyte [16], we propose a new model, “Epm”, that includes synaptic input and is more robustly excitable. Over the range 200–600 Hz, Epm predicts that ATP consumption per AP is not constant. Using Epm to explore the source of Na^+^-entry at various frequencies leads to the prediction that Nav channel density but not AChR channel density should increase as a function of AP (i.e. EOD) frequency. In the context of the EOD-linked O_2_-consumption reported by Lewis et al [6], these results strongly suggest that the electrocytes themselves are energetically efficient devices and that a greater fraction of global cost of EOD production can be attributed to pre-synaptic processes (e.g., packaging and recycling of ACh quanta) than to supporting electrocyte ion homeostasis. Comparable predictions have been made, interestingly, regarding the relative costs incurred for synaptic transmission versus electrical propagation by in situ mammalian central neurons [3, 4, 17].

## Methods

### Limitations of a previous model

Since *Eigenmannia* electrocytes’ continual APs demand narrowly-maintained equilibrium potentials (E_K_ and E_Na_), homeostatic processes evidently succeed in minimizing fluctuations of [Na^+^] and [K^+^] close to the AP-generating membrane. A previously developed excitability model for isolated electrocytes (termed here, “MKZ” for Markham, Kaczmarek, Zakon, 2013) [16] depicts a system that is quiescent until stimulated by intracellular current injection (I_clamp_). This model does not include an explicit Na^+^/K^+^ pump so initially we intended to incorporate one and explore E_K_ and E_Na_ stability for APs at frequencies over the species range (200–600 Hz). Because pump function is electrogenic, a key system requirement is to stabilize membrane potential (V_m_) at the resting value (V_rest_) of the unstimulated excitable system. Stabilization is accomplished via Na^+^ and K^+^ leaks of appropriate magnitudes [18] This however renders MKZ inexcitable because the very small V_*rest*_-E_K_ necessitates an enormous K^+^-specific leak to counter pump electrogenicity and this makes V_m_ ultra-stable near V_rest_.

**Fig 1C** shows MKZ APs during a 200 Hz stimulus train (same parameters as in Fig 3A in Ref. [16]); an expanded AP (right panel) reveals that the regenerative ΔV_m_ covers ~35 mV (from ~-17 mV to +17 mV) and that V_peak_ depends strongly on I_stim_ (see inset) and so is not strictly all-or-none. Excitability that depends strongly on large amplitude stimuli in this way seems ill-suited for *Eigenmannia*’s robust high frequency EOD output.

The MKZ model assumes an isopotential electrocyte in which the membrane current (I_membrane_) charges and discharges 50 nF of membrane capacitance. While space-clamp (isopotential) is necessary for electrocyte voltage clamp recordings, electrocytes are in general not isopotential in situ as Na^+^-entry occurs exclusively via Nav channels at the innervated posterior membrane, located ~1 mm distant from the anterior membrane, which is channel-rich but inexcitable [5] In *Sternopygus* [19] and presumably *Eigenmannia* current flow across the intracellular series resistance results in an anterior-posterior potential difference of several tens of millivolts.

An excitability model applicable to EODs should mimic AP features of healthy electrocytes under current clamp (I_clamp_) where AP thresholds are close to -45 mV during tonic firing when evoked by a constant current stimulus (40 ms) [16]. Applying constant current stimuli to the MKZ model (rather than the much larger 0.2 ms current pulses used by Lewis et al. [6] however, elicits only one high threshold AP and after a damped oscillation, the V_m_ settles to a depolarized plateau. Inspection of **Fig 1C** shows that for modeled MKZ-APs elicited by a 0.2 ms pulse, most of the depolarization (~60 mV of the total AP voltage excursion) is accomplished not by I_Na_ but by the large injected current. The model developed in the next sections, Epm, addresses this and several other limitations of the MKZ model.

The new excitability model (Epm) considers only the posterior excitable membrane. Demand on the Na^+^/K^+^ pump resulting from APs is inferred from net Na^+^-entry across this membrane. No specific inference is made about where and how pump electrogenicity is managed in the electrocyte. This issue, plus anterior membrane contributions to high-frequency EODs and ion homeostasis will be addressed in later work.

APs are spikes in the transmembrane potential, *V* (or *V*_*m*_), and are solutions of the equation:

$$C\frac{dV}{dt}=-I_{NaT}-I_{NaP}-I_{K}-I_{L}+I_{stim},$$ (1)

where *C* is the capacitance of the electrocyte membrane, *I*_*NaT*_ (transient) and *I*_*NaP*_ (persistent) are the currents through Nav channels, *I*_*K*_, the current through Kv channels, *I*_L_, the generic leak current and *I*_*stim*_, the stimulus current initiating the APs. We only show the currents included in the Epm model. The reader is referred to [16] and [6] for other currents in the MKZ model.

$$I_{NaT}={\mathrm{gNa}}_{\max}m^{3}h(1-\gamma )(V-E_{Na}),$$ (2)

$$I_{NaP}={\mathrm{gNa}}_{\max}m^{3}\gamma (V-E_{Na}),$$ (3)

$$I_{K}={\mathrm{gK}}_{\max}n^{4}(V-E_{K}),$$ (4)

where the kinetic variables *j = m*, *h*, and *n* obey the equation:

$$\frac{dj}{dt}=\alpha_{j}(1-j)-\beta_{j}j,$$ (5)

while *I*_*L*_ is given by:

$$I_{L}=g_{L}(V-E_{L}),$$ (6)

and *I*_*stim*_ can flow either through a current clamp electrode or through post-synaptic cation channels, as presented in the next section. Values for parameters in the models MKZ and Epm appear in **Table 1**.

**Table 1:** Kinetic constants for the MKZ and Epm models. MKZ parameters are from Ref [6]. Listed gNa_max_ values are for the 200 Hz case. For Epm, the gNa_max_ values used for other frequencies are discussed in the Section **Frequency-dependent cost per AP**.

	MKZ	Epm
*g*_L_	0.76 μS	5 μS
*C*	50 nF	50 nF
**Equilibrium and reversal potential**		[Na]_i_ = 1.35 mM
		[Na]_o_ = 120 mM
		[K]_i_ = 89 mM
		[K]_o_ = 2.16 mM
*E*_*Na*_	50 mV	55 mV
*E*_*K*_	-95 mV	-94 mV
*E*_*L*_	-93 mV	*E*_K_
*V*_rev_(AChR)	n.a.	2.18 mV
***g***_***Na***_***(V)* Na channels**		
gNa_max_	700 μS	700 μS
*γ*	0.1	0.02
*k*_αm_	7.6 ms^-1^	8.03 ms^-1^
*k*_βm_	0.6894 ms^-1^	0.2195 ms^-1^
*η*_*αm*_	0.0037 mV^-1^	0.0037 mV^-1^
*η*_β*m*_	-0.0763 mV^-1^	-0.0763 mV^-1^
*k*_αh_	0.00165 ms^-1^	0.02247 ms^-1^
*k*_βh_	0.993 ms^-1^	n.a., HH type
*η*_*αh*_	-0.1656 mV^-1^	-0.06802 mV^-1^
*η*_β*h*_	-0.0056 mV^-1^	n.a. (see *)
***g***_***K***_***(V)* delayed rectifier**		
gK_max_	2000 μS	2000 μS
*k*_αn_	1.209 ms^-1^	2.135 ms^-1^
*k*_βn_	0.4448 ms^-1^	0.3524 ms^-1^
*η*_*αn*_	0.00948 mV^-1^	0.03792 mV^-1^
*η*_βn_	-0.01552 mV^-1^	-0.01552 mV^-1^
*a* *	2.5 mM^-1^ ms^-1^nA^-1^	n.a.
*a'* *	12.5 mM^-1^ ms^-1^	n.a.
*b* *	0.5 ms^-1^	n.a.
*k*_*f*_ *	10 mM^-1^ ms^-1^	n.a.
*k*_*b*_ *	2.0 ms^-1^	n.a.
**Inward rectifier**	(*g*_*KIR*_)	
*g*_*R*_	300 μS	0
*η*_*R*_	0.22 mV^-1^	n.a.
**AChR (post-synaptic)**		
*P*_Na_	n.a.	0.00016 mm^3^/s
*P*_K_	n.a.	1.11 *P*_Na_

* In the MKZ model the impact of intracellular Na on the Kv conductance is modeled through a parameter *s* as described in Eqs 10 and 11 in Ref. [6] and on p. 1715 of Ref. [16]. The * parameters characterize the equations governing the kinetics of *s* which turns out to have no effect on Kv conductance because, after 1 ms, *s* deviates very little from unity.

Further details on the above currents are discussed in the following sections starting with the cation channel version of *I*_*stim*_.

### A post-synaptic cation conductance for Epm

EOD frequency-dependent O_2_ consumption [6] pertains to the *in vivo* situation, with APs triggered by post-synaptic current through acetylcholine receptor channels (AChRs). In Epm, an electro-diffusion description of ion permeation through this class of channel allows for separate tallying of Na^+^ and K^+^ fluxes.

$$I_{stim}={\mathrm{syn}}_{clamp}(t)(I_{AChR(Na)}+I_{AChR(K)})$$ (7)

with:

$$I_{AChR(X)}=P_{X}Fz\left(\frac{zFV/RT}{1-e^{-zFV/RT}}\right)\left[{\left[X\right]}_{in}-{\left[X\right]}_{out}e^{-zFV/RT}\right],$$ (8)

where *X* is either Na^+^ or K^+^, the two ions that are permeant through the AChR channels, *F* is the Faraday constant, *R* the gas constant, *V* the transmembrane potential and *z* the valence of the ions. The ratio of the permeabilities *P*_*K*_/*P*_*Na*_ is 1.11 as given in **Table 1**and the absolute value of *P*_*Na*_ was chosen to yield an appropriate-sized stimulus for the modeled posterior membrane. In the standard pulsatile stimulation used here the maximum value of syn_clamp_*(t)* is 1; higher values will imply correspondingly higher densities of activatable AChRs. In what follows syn_clamp_*W* or syn_clamo_W_0Hz_ will indicate a constant “cation permeability clamp” of magnitude W (for example syn_clamp_*0*.*5* means that syn_clamp_*(t)* = *0*.*5*).

For *Sternopygus* [20] and *Electrophorus* [21] excitatory postsynaptic potentials (epsp) and miniature post-synaptic currents at the “electroplax” (mepsc), respectively, have been recorded. *Electrophoru*s miniatures are slower than those at frog endplates [21], nevertheless, during predatory strikes, the electric eel can produce brief EOD bursts at ~400 Hz [22] (mechanistic details are unknown). *Eigenmannia* EODs demand continuous APs at up to 600 Hz [23] presenting an even more extreme challenge, assuming the electrocytes fire 1:1 with the EOD frequency [14, 19]. Even a subset of exceptionally short-lived events recorded from *Torpedo* electroplax (an elasmobranch, not a teleost) [24] or the fast rising endplate currents reported for lizard muscle [25] would be too slow overall. We model high frequency synaptic AChR activation using a pulsatile syn_clamp_ stimulus that could operate effectively up to just beyond 600 Hz. Because such short-lived stimuli are strictly conjectural, we also consider steady-state (0 Hz) syn_clamp_, and mixed stimuli (i.e. pulsatile atop some background AChR activation).

### gNa(V) characteristics for Epm

The voltage dependent kinetic variables α_j_ and β_j_ in Epm (and MKZ) have the form $\alpha_{j}=k_{\alpha j}e^{\eta_{\alpha j}V}$ or $\beta_{j}=k_{\beta j}e^{\eta_{\beta j}V}$ where the pre-factors *k*_*αj*_ and *k*_*βj*_, and the exponential factors *η*_*αj*_ and *η*_*βj*_ are given in **Table 1**. The one exception is the kinetic variable *β*_*h*_ which describes the rate of inactivation. In Epm it has the functional form used by Hodgkin and Huxley for squid axon:

$$\beta_{h}=\frac{3.33}{\exp(-(V+30)/9)+1.0}.$$ (9)

**Fig 2:**
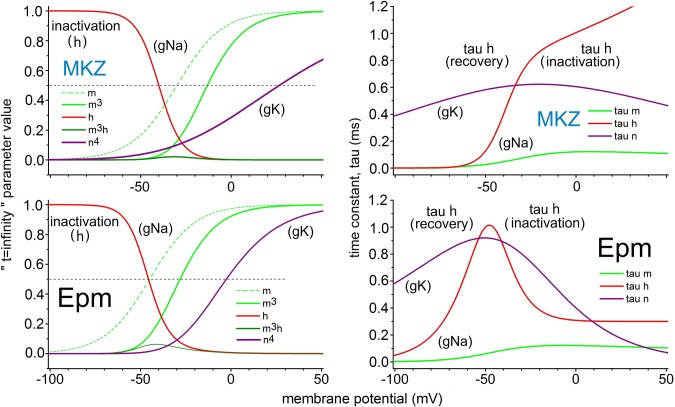
Graphical comparison of the V-gated conductances of MKZ and Epm. The plots are generated using parameters in **Table 1**. Left: equilibrium (i.e., *t* → ∞) values of the Hodgkin-Huxley parameters. As labeled, gNa and gK are proportional to m^3^ and n^4^ respectively, and, h is proportional to the availability (or inactivation status) of the Na conductance. Right: time constants for these processes (see text for more explanation). For tau_h_ (V) curves, the voltage regions dominated by the recovery and the inactivation processes are indicated.

For MKZ and Epm, **Fig 2**plots the characteristics in Hodgkin-Huxley terms [26, 27] of the voltage-gated conductances. In MKZ the *m*^*3*^*(V)* midpoint is -13 mV (**Fig 2**). The G/G_max_(V) reported for *Eigenmannia* electrocytes is, however, -37 mV (Fig 3 of Markham et al., 2013 [16]); for Epm the choice of prefactor *k*_*αm*_ set the midpoint of *m*^*3*^*(V)* at -28mV (see **Fig 2**). For the rate of recovery from inactivation (forward inactivation rate) *α*_*h*_ *(V)* (**Eq (3)**), the exponential factor *η*_*αh*_ has the value used by Cannon and colleagues (Table 1 in [28]; originally from Pappone [29] for mammalian skeletal muscle).

$$\alpha_{h}=k_{\alpha h}e^{\eta_{\alpha h}V}$$ (10)

The prefactor *k*_*αh*_ was adjusted to yield a robust window conductance (see *m*^*3*^*h* for Epm in **Fig 2**). Our choice of the backward inactivation rate *β*_*h*_*(V)*(**Eq (9)**) ensures the fast development of the APs and a very brief refractory period. We note that while in the Hodgkin-Huxley papers, *τ*_*h*_ (for the squid axon) converges to 1 ms for large depolarizations, the parameters here make *τ*_*h*_ go asymptotically to 0.3 ms as reported by Shenkel and Sigworth [30] (see their Fig 3B up to ~+20 mV and their Fig 8C) for *Electrophorus* electrocytes. Epm inactivation kinetics, unlike in MKZ, conform to a standard Hodgkin-Huxley form (see τ_h_ plots, **Fig 2**). In the electric fish *Electrophorus*, *Sternopygus*, and *Eigenmannia*, *τ*_*h*_ begins to increase at large depolarizations [16, 20, 30], but because the voltages in question are beyond the physiological range, this feature was not included in the model.

**Fig 3:**
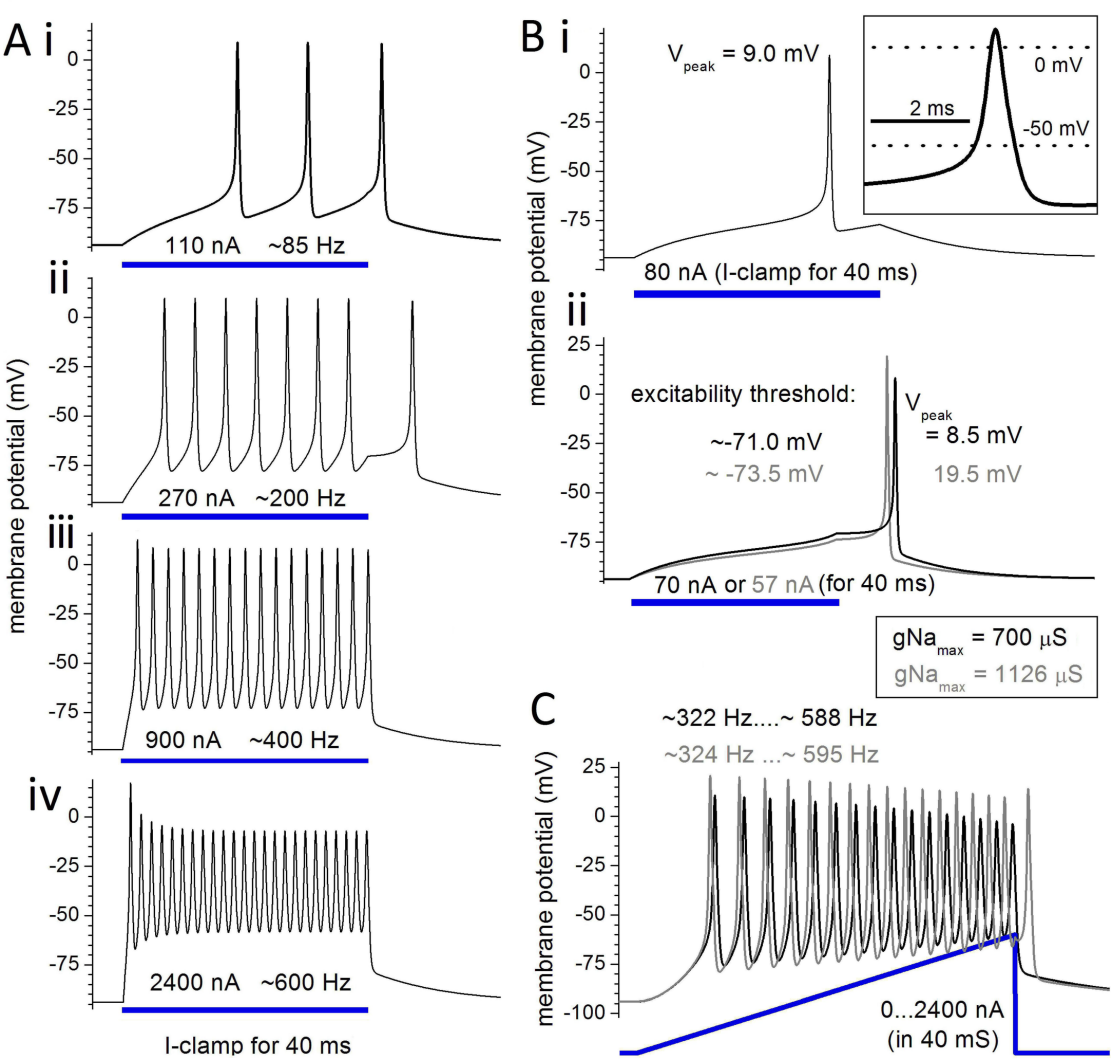
Sustained step and ramp I_clamp_ stimulation of Epm. Standard gNa_max_ (700 μS) is used unless otherwise specified, I_stim_ as labeled, other parameters as in **Table 1**. See text for further explanation.

Epm retains a simple persistent gNa(V) with the same activation kinetics as for the transient gNa(V) and no inactivation (see Eq (3). It has a lower magnitude than MKZ, i.e., γ = 0.02 in Epm versus 0.1 in MKZ (**Table 1**)).

### K conductances for Epm

It was formerly thought that Na^+^-activated *I*_*K*_ (*I*_*KNa*_) and persistent *I*_*Na*_ recorded from *Eigenmannia* electrocytes form an interacting pair [16], but subsequent immunocytochemical evidence located *K*_*Na*_ and Nav channel proteins exclusively to anterior and posterior membranes, respectively [5]. While Epm retains a (diminished) persistent gNa, it does not include the gK_Na_. Moreover, its description in MKZ as a “Na^+^-activated gK” notwithstanding, this conductance operates as a classic delayed rectifier under the modelled conditions. Its modulation by *s*, the non-dimensional parameter sensitive to [Na^+^]_intracellular_ (see *Materials and Methods* section in [16]), is inconsequential since the value of *s* converges within ~ 1 ms to unity. Additionally, ∆[Na^+^]_intracellular_ had no impact on *E*_*Na*_ which is held constant in that model. Thus with or without the MKZ “*Na*-modulation” (asterisks (*) in **Table 1**), MKZ APs (e.g. **Fig 1C**) are identical. Accordingly, additional, experimental and computational, efforts are needed to resolve the functional contributions of *gK*_*Na*_ in *Eigenmannia* electrocytes.

*Eigenmannia* electrocytes have an inwardly rectifying *gK* [16] whose normal operating range is sufficiently hyperpolarized that even if some were present at the posterior membrane, it would neither contribute to nor interfere with excitability. Skeletal muscle t-tubular membrane [31] has abundant Kir2 protein. Kir2 mRNA transcripts have been detected in *Eigenmannia* electrocytes [32], but these channels have not to date been localized in the electrocyte, whereas Kir6 protein (an ATP-sensitive *gIR*) is expressed only on the anterior electrocyte membrane [5]. Thus, MKZ included *gIR*, but Epm does not.

Although a delayed rectifier current is evident in *Sternopygus* [20], a Na^+^-independent delayed rectifier K^+^ current is not present in *Eigenmannia* electrocytes [16]. However, without a *gK(V)*, high frequency excitability fails. It has been suggested for *Electrophorus* that “the membrane potential is repolarized by Na^+^ channel inactivation, and from the conductance of Cl^-^ and K^+^ through leak channels” (p. 230 in [33]), in other words, without a delayed rectifier. This seems untenable for *Eigenmannia* whose electrocytes must fire from 200 Hz up to 600 Hz. With no delayed rectifier, the 50 nF membrane would need an RC time constant of ~1 ms to repolarize by ~ -100 mV (+20 mV to -80 mV). This requires a leak (*g*_*K*_ and/or *g*_*Cl*_) of 50 μS (for reference *g*_*Leak*_ = 0.76 μS in MKZ) that would render the membrane inexcitable unless gNa_max_ was massive. For Epm, we retained the *C*_*m*_ = 50 nF of MKZ, thus requiring the inclusion of a delayed rectifier K^+^ conductance in Epm. For this delayed rectifier we retained the MKZ gK_max_ of 2000 μS. With these constants, use of gNa_max_ = 700 μS gives a reasonable overshooting V_peak_ value for APs at the low end of the EOD range (200 Hz) (e.g. use of at gNa_max_ = 400 μS at 200 Hz gives V_peak_ = ~0 mV). Accordingly, for Epm, gNa_max_ = 700 μS was made the “standard” for APs at 200 Hz (**Table 1**).

Having settled on the parameters for gNa(V) and established the need for a delayed rectifier (gK(V)) within Epm, it was necessary to design the latter to allow for APs up to 600 Hz. We assumed that gK(V), when activating, should not interfere with the depolarizing I_Na_, but instead should develop rapidly just after the AP has overshot 0 mV, and thus drive repolarization. An I_K_(V) that activates in this manner would be consistent with an impedance analysis of *Sternopygus* electrocyte APs (see Fig 28 in [19] or Fig 41 in [34]). That analysis shows that after a conductance associated with rapid depolarization falls rapidly, another conductance grows rapidly. These characteristics were achieved via the Epm gK(V) parameters listed in **Table 1,** which result in a gK activation curve (*n*^*4*^(V) in **Fig 2**) whose midpoint is -2.6 mV and whose slope is 0.017 mV^-1^ (equivalent to 14.7 mV per e-fold). Interestingly, these values are very similar to those reported for a skeletal muscle gK(V) (Kv3.4, Fig 2C of [35]).

## Results

### Epm responses to constant current stimulation

For Epm, a sustained constant current stimulation (I_stim_) (40 ms, as per *Eigenmannia* I-clamp experiments: Fig 1 of [16]) yields trains of overshooting, all-or-none, strongly regenerative APs that, depending on I_stim_ intensity, fire at frequencies from below to above the species EOD range (200–600 Hz) (**Fig 3Ai-iv**). Even after I_stim_ ends Epm can fire a full-amplitude AP before settling to V_rest_ (**Fig 3Aii**). Note that as I_stim_ is increased the frequencies near 600 Hz (**Fig 3Aiv**) can be attained but at diminished AP amplitude. Another indicator that Epm is robustly excitable is seen in **Fig 3B**, where an AP fires as the membrane depolarizes past ~-70 mV during a small I_stim_ (**Fig Bi**) or even after, provided the I_stim_ value is extremely close to threshold (**Fig Bii**). For larger values of gNa_max_ (grey in **Fig 3Bii**), minor quantitative differences in AP characteristics are evident. The model electrocyte would be even more prone to fire in the presence of the current noise typical during synaptic activation. The Epm system exhibits “Class 1 excitability” (pp 219–221 of [36]); APs of arbitrarily low frequency can be triggered, depending on the strength and duration of I_stim_.

In **Fig 3C,** firing is elicited by an I_stim_ ramp of 40 ms duration going to a maximum of 2400 nA, resulting in frequencies that begin near ~320 Hz and increase to ~600 Hz (note the progressively diminishing AP amplitude for both gNa_max_ = 700 and 1126 μS). The initial frequency depends on ramp steepness (the shallower the slope the lower the initial frequency, not shown) and the maximal frequency, which is set by channel kinetics, depends only on I_stim_ magnitude.

Reduced V_peak_ values at high frequencies (e.g., see **Fig 3Aiv** and **3C**) point to a question addressed in the next section and raised previously [6], namely, is there a need for higher Nav channel density to sustain higher frequency APs? For *Eigenmannia* individuals this would correspond to a positive correlation between EOD frequency and Nav channel density.

### Epm responses to pulsatile synaptic current stimulation

**Fig 4:**
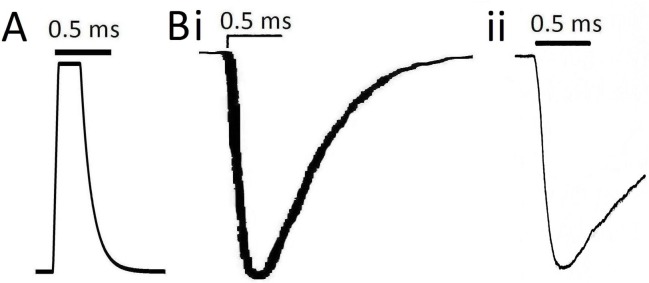
**A.** The standard pulsatile stimulus shape syn_clamp_ (t) used in Epm has 3 segments: a linear rise for 0.05 ms, a plateau for 0.200 ms and an exponential decay to zero (specifically the segments are: syn_clamp_1.0 (t) = t/0.05 (t<0.05); 1 (0.05<t<0.25); and exp[-(t-0.25)/0.1] (t>0.25), t in ms)). **Bi.** For comparison, a depiction of the fastest cholinergic post-synaptic event we found in the literature for electroplaques, shown at the same time scale as the standard pulsatile stimulus in A. The miniature electroplaque current (mepc) depicted exemplifies a fast subclass of of mepc recorded in relatively intact *Torpedo* electroplaques [24]. **Bii** depicts the average time course of mepcs measured from lizard intercostal muscle [25]. The pulsatile stimulus used in Epm is speedier in all respects than these mepcs. Whereas a mepc results from a quantal release event that would be insufficient to stimulate an entire electroplaque, the syn_clamp_ stimuli of Epm must provide sufficient “ACh-activated” I_cation_ at the *Eigenmannia* electroplaque to rapidly depolarize to threshold 50 nF of post-synaptic membrane. Thus, 1:1 triggering (1 electromotor neuron AP: 1 electrocyte AP) is assumed, but how it would be achieved at up to 600 Hz in vivo is not understood.

We assume that each electrocyte AP is triggered by a brief pulsatile AChR-mediated post-synaptic current intense enough to yield full-amplitude APs at frequencies up to 600 Hz (see **Fig 4**). The shape of this standard stimulus (syn_clamp_1) is based on synaptic currents in Torpedo [24] and lizard [25] see **Fig 4**legend and **Table 1**.

**Fig 5:**
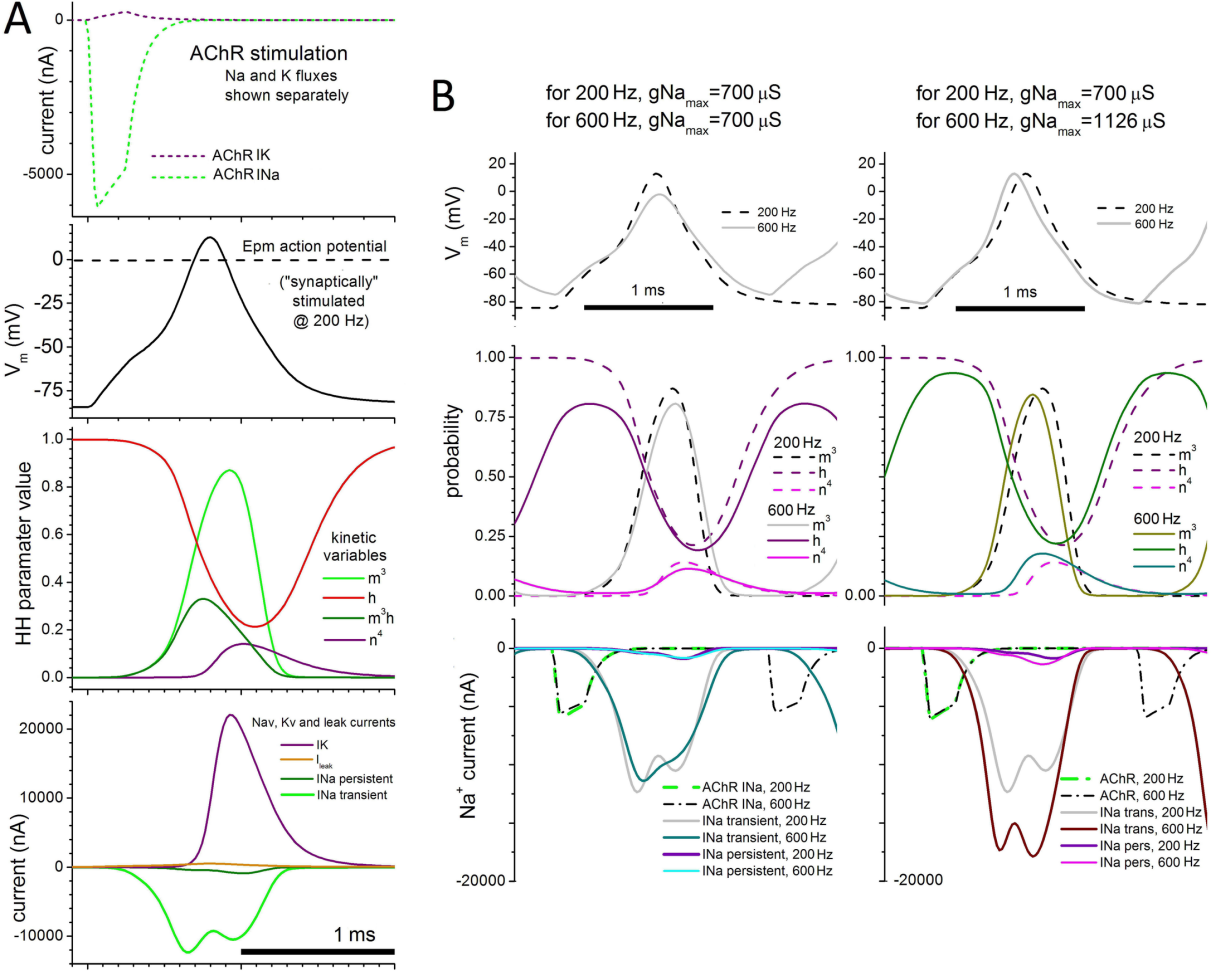
Epm-simulated APs stimulated by pulsatile synaptic current. **A.** One AP from a 200 Hz AP train (stimulus: syn_clamp_1.0_200Hz_) with gNa_max_ = 700 μS. **B.** For gNa_max_ values as labeled, APs from trains with stimulus syn_clamp_1.0_200Hz_ or syn_clamp_1.0_600Hz_. First column, gNa_max_ = 700 μS; second column, for syn_clamp_1.0_600Hz_, gNa_max_ is raised to 1126 μS to bring V_peak_ to 13 mV. See text for further explanation.

**Fig 5A** plots the membrane potential and underlying variables for a single Epm AP in a 200 Hz train. During the stimulus (top) net Na^+^ influx (I_Na_) is substantial while I_K_ is minimal (E_K_ is near V_rest_). Because the stimulus ends before V_m_(t) reaches the V_rev_ for AChRs (+2.2mV) both I_K_ and I_Na_ components of the syn_clamp_ remain unidirectional. As in I-clamped *Eigenmannia* electrocytes [16] the AP shows an apparent threshold region near -45 mV (**Fig 5A**). The AP peaks at ~13 mV (for computations V_peak_ = +12.86 mV), a value used in connection with syn_clamp_1 as the “standard” AP-amplitude. Early I_Na_(V,t) is unopposed but the rapid-onset of I_K_(V,t) then drives AP repolarization in co-operation with gNa inactivation. Recovery from inactivation is almost complete ~1 ms after V_peak_. As a result Epm APs at 200 Hz are indistinguishable from APs at lower frequencies.

**Fig 6:**
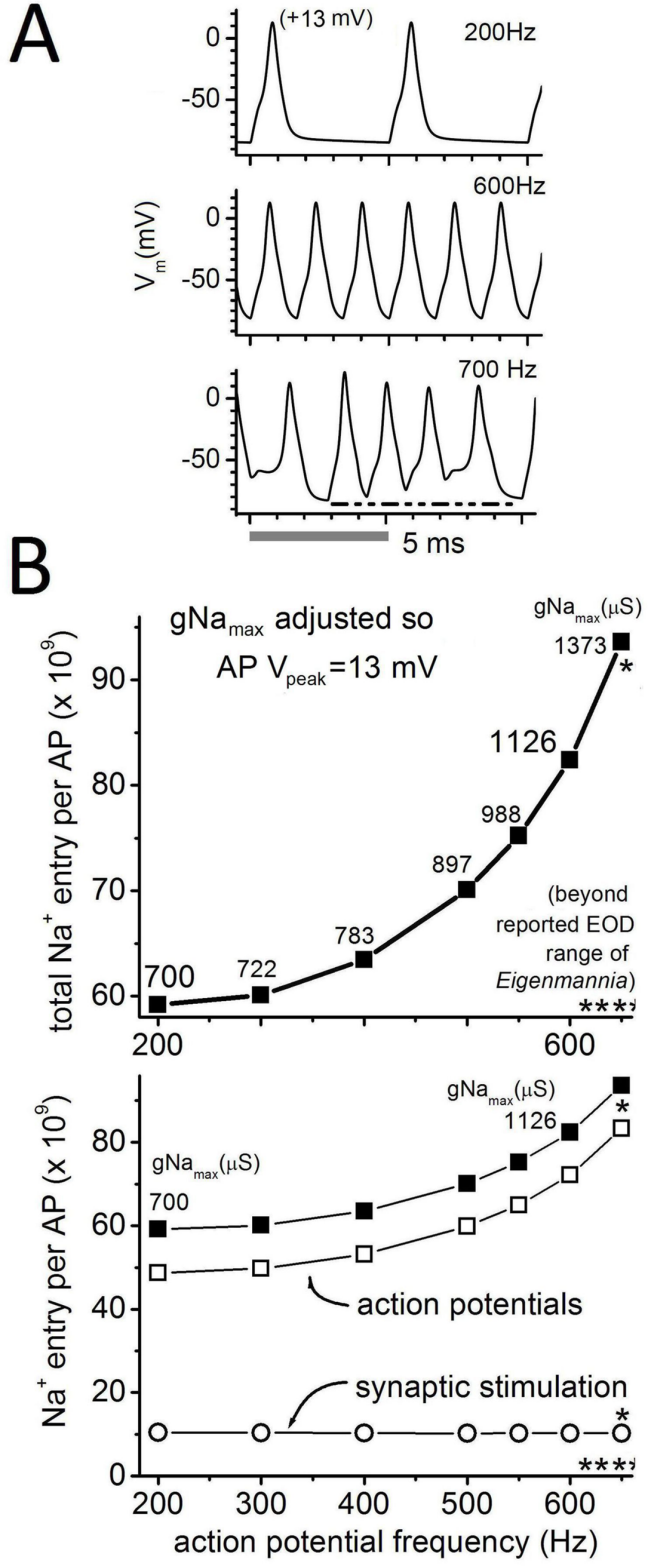
Cost of Epm APs at different AP frequencies based on Na^+^-entry. **A.** Illustrated are APs firing at 200 Hz and 600 Hz and then failing at 700 Hz which, however, exceeds the biological range of *Eigenmannia* (700 Hz stimulation elicits APs with irregular amplitudes and timing). For all frequencies the pulsatile stimulus amplitude was syn_clamp_1.0 and gNa_max_ was adjusted to yield V_peak_ = 13 mV. To calculate Na^+^-entry (= the time integral of the three sources of I_Na_ seen in **Fig 5A**) trains of at least 20 APs were used. **B.** Na^+^ entry plotted to assess the cost/AP at different frequencies, as labeled and as explained in the text. Starred region beyond 600 Hz signifies that although this is beyond the species range, Epm is still able to produce regular APs at 650 Hz (though not, as seen in **B**, at 700 Hz). Calculations were done by requiring that V_peak_ = 12.86 mV for each frequency but this is referred to throughout the paper as 13 mV. Larger fonts for gNa_max_ values at 200 Hz and 600 Hz emphasize that these represent the extremes of the biological range for *Eigenmannia* EODs.

To maintain a constant V_peak_ at higher frequencies, gNa_max_ values must increase (as with MKZ; [6]). **Fig 5B** compares model variables at 200 Hz and 600 Hz with gNa_max_ kept at 700 μS for both frequencies (left) or with gNa_max_ increased to 1126 μS for the 600 Hz APs (right). These values plus those needed for intervening frequencies are also indicated as plot labels in **Fig 6B.** The bottom panels of **Fig 5B** show that Na^+^ entry at 200 Hz vs 600 Hz is unaffected if the AP amplitude (top panels) is allowed to fall (left) but increases markedly if gNa_max_ is adjusted to attain the standard V_peak_ (top right). The middle panels reveal that the voltage-gated channels’ activation kinetics are not limiting here; the rise and fall of parameters m^3^ and n^4^ (which reflect open-gNa and open-gK) is rapid enough at both 200 and 600 Hz (middle panels).

Because gK_max_ was held constant across frequencies while gNa_max_ was increased, the AP depolarization rate and hence the speed of gNa activation and inactivation onset increases with frequency. The limiting factor for maintaining full amplitude APs is recovery from inactivation. The middle panels of **Fig 5B** show that *h* (sodium channel availability or inactivation status) at 600 Hz has insufficient time to return to unity after each AP; at 600 Hz *h* barely reaches 0.8 before the next AP. Failure to fully recover from inactivation underlies the requirement for increased gNa_max_ at 600 Hz. Thus, while overall AP shapes differ only subtly between 200 and 600 Hz, the faster rise to threshold (and then to peak) at 600 Hz is not inconsequential. It is possible only because of the augmented Na^+^-entry (**Fig 5B** bottom panel, right).

### Frequency-dependent cost per AP

Thus, in the Epm model, the increased gNa_max_ needed to sustain APs at full amplitude with increasing frequency predicts that the cost/AP (Hz) will rise. **Fig 6A** illustrates a few APs from trains of Epm APs elicited by pulsatile syn_clamp_ at 200 Hz, 600 Hz, and the unphysiologically rapid 700 Hz. Such APs are used to calculate Na^+^-entry (trains of at least 20 APs were generated), which is a proxy for energetic cost/AP. Given sufficient gNa_max_, AP trains are attainable up to ~650 Hz in Epm, whereafter irregularities appear (e.g., see 700 Hz in **Fig 6A**).

The upper panel of **Fig 6B** combines Na^+^ entry through V-gated (Nav) and synaptic (AChR) channels. The cost/AP increases nonlinearly over this range of frequencies. However, separating the synaptic cost/AP(Hz) reveals that the cost of synaptically stimulating APs is essentially constant. Hence, the non-linearity in cost/Hz can be ascribed to Nav currents. Since our initial choice of gNa_max_ = 700 μS at 200 Hz may seem arbitrary, we also ran calculations with gNa_max_ = 900 μS. Briefly, this increases V_peak_ to ~18 mV, which means larger values of Na^+^ entry for the gNa, but nearly unchanged synaptic costs.

### Is all depolarizing Na^+^-entry equivalent?

**Fig 7:**
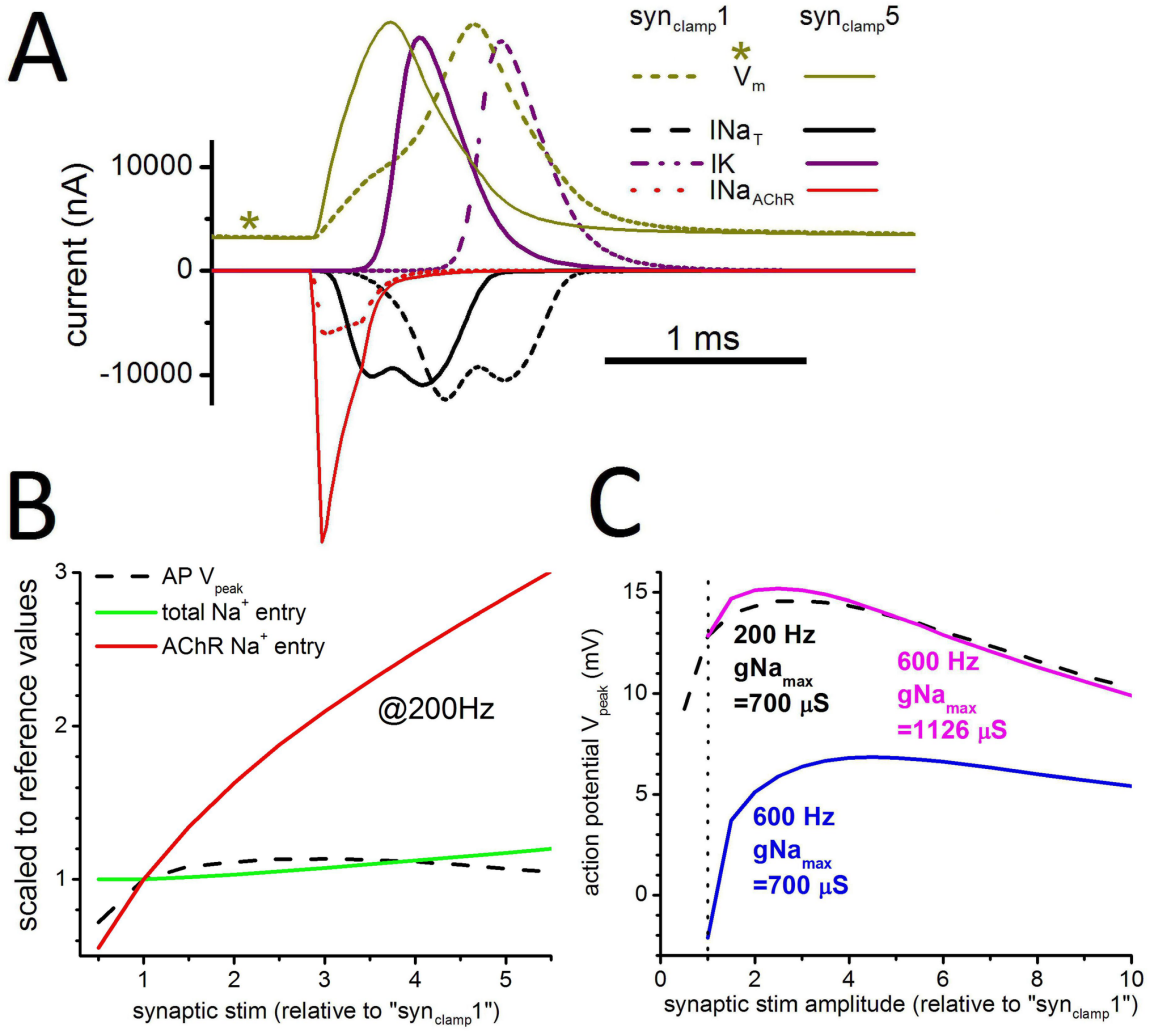
Effect of pulsatile stimulus amplitude. **A.** Single APs elicited by the standard pulsatile stimulus (V_m_(t) units are arbitrary, with the syn_clamp_1_200Hz_ AP as reference) calculated for syn_clamp_1_200Hz_ or syn_clamp_5_200Hz_, and V-gated currents (INa_T,_ where T signifies the transient component; persistent INa not shown), IK (delayed rectifier) plus the INa component of current through the AChR channels; gNa_max_ = 700 μS, stimuli at 200 Hz. **B.** For such APs elicited by pulsatile synaptic stimulation at increasing intensities (from syn_clamp_0.5_200Hz_ to syn_clamp_5.0_200Hz_), Na^+^-entry via the stimulus (AChR) channels increases dramatically (red) but total Na^+^-entry (green) is almost unaffected. The V_peak_ is relatively insensitive to stimulus intensity beyond syn_clamp_ = 1 (an “all-or-none” AP feature). **C.** Further testing of pulsatile syn_clamp_ intensity, for APs at 200 Hz and 600 Hz with the gNa_max_ values indicated. The dashed black line in **C** is equivalent to the dashed black line in **B** (where values are normalized).

If synaptic stimuli with amplitudes different from syn_clamp_1 are used, does this picture change? **Fig 7**examines this question, with **Fig 7A** showing V_m_(t), plus the key attendant currents for a single AP triggered by pulsatile (200 Hz) syn_clamp_1 or syn_clamp_5 (a value 5 times larger than syn_clamp_1). As expected, the AP rise time is faster with the stronger synaptic input, but the I_Na_ and I_K_ profiles are similar for both inputs. **Fig 7B** provides a comparison over this range of stimulus amplitudes. The black dashed-line shows that syn_clamp_1 triggers APs of V_peak_ = 13 mV and that V_peak_ changes little with larger stimuli. And strikingly, the larger stimuli hardly alter the total Na^+^-entry cost. Even though the increased stimulus (“more clamped-open AChRs”) is allowing more Na^+^-entry by this route (see the strong rise of the red line), total Na^+^-entry (green line) into the electrocyte increases only slightly. With much more of the depolarization accomplished early due to Na^+^ influx through AChR channels, the need for influx through Nav channels once they activate is diminished.

Would this outcome signify that a fish could be energetically “indifferent” to which class of channels carry the major load for depolarization? We suggest not. A cost analysis based solely on redressing Na^+^-entry into electrocytes ignores metabolic costs incurred pre-synaptically. Costs related to producing and packaging AChR molecules is not captured by the Na^+^-entry tally. Biologically, therefore, if EOs are driven by pulsatile synaptic stimuli, operating near our reference state (i.e. coordinates 1,1 of **Fig 7B**) would be optimal, ensuring signal integrity while avoiding high presynaptic costs.

Whereas **Fig 7B** deals only with APs at 200 Hz, **Fig 7C** looks also at 600 Hz. Both **Fig 7B** and **7C** have dashed black lines for the 200Hz V_peak_, but note that the Y-axis in **Fig 7C** is the absolute value of V_peak_. Note too that for X-axis position “syn_clamp_1” in **Fig 7C**, for each of the 3 plot lines, the relevant APs would be those from **Fig 5B** (top panels)**.**

The question here: to produce a full sized V_peak_, could larger pulsatile stimuli (up to 10X syn_clamp_1) compensate for an otherwise too-low gNa_max_? The consequences of larger stimuli are qualitatively the same across the frequency range, as shown in **Fig 7C** which varies syn_clamp_ for 200 Hz and 600 Hz, with appropriate gNa_max_ values (see mauve and black dashed lines; the blue plot simply demonstrates that gNa_max_ appropriate to 200 Hz makes full amplitude APs at 600 Hz impossible for any stimulus intensity). Near syn_clamp_2, AP peaks are slightly more depolarized than for syn_clamp_1 and all-or-none behavior is particularly robust, with stimulus intensity variations of ~±0.3 in yield essentially the same AP amplitude. Rightward in the plots, V_peak_ values fall; larger syn_clamp_ becomes counterproductive.

Would the somewhat more robust all-or-none behavior near syn_clamp_2 make it preferable to syn_clamp_1? Not necessarily, since synaptic transmitter costs would nearly double. Taken together, Epm predicts (based on the three curves of **Fig 7C**) that across *Eigenmannias*’s 200–600 Hz EOD range, adequately large APs would be attained via appropriately-sized gNa_max_, not by augmenting stimulatory AChR currents. Similarly, variations in gK_max_ cannot maintain V_peak_ across these frequencies. A Nav channel density adequate for the low end of the *Eigenmannia* EOD frequency range could not be compensated for in high frequency fishes by adjusting gK_max_ (data not shown). This leads to the broad prediction that AChR density would be similar for fish operating at low and high frequencies, while Nav channel density would increase for higher frequency fish. With <2-fold difference in gNa_max_ predicted for 200 Hz vs 600 Hz fish, however, experimentally quantifying such moderate density differences may be challenging.

### Simulating electrocyte action potentials during rapid changes in EOD frequency

**Fig 8:**
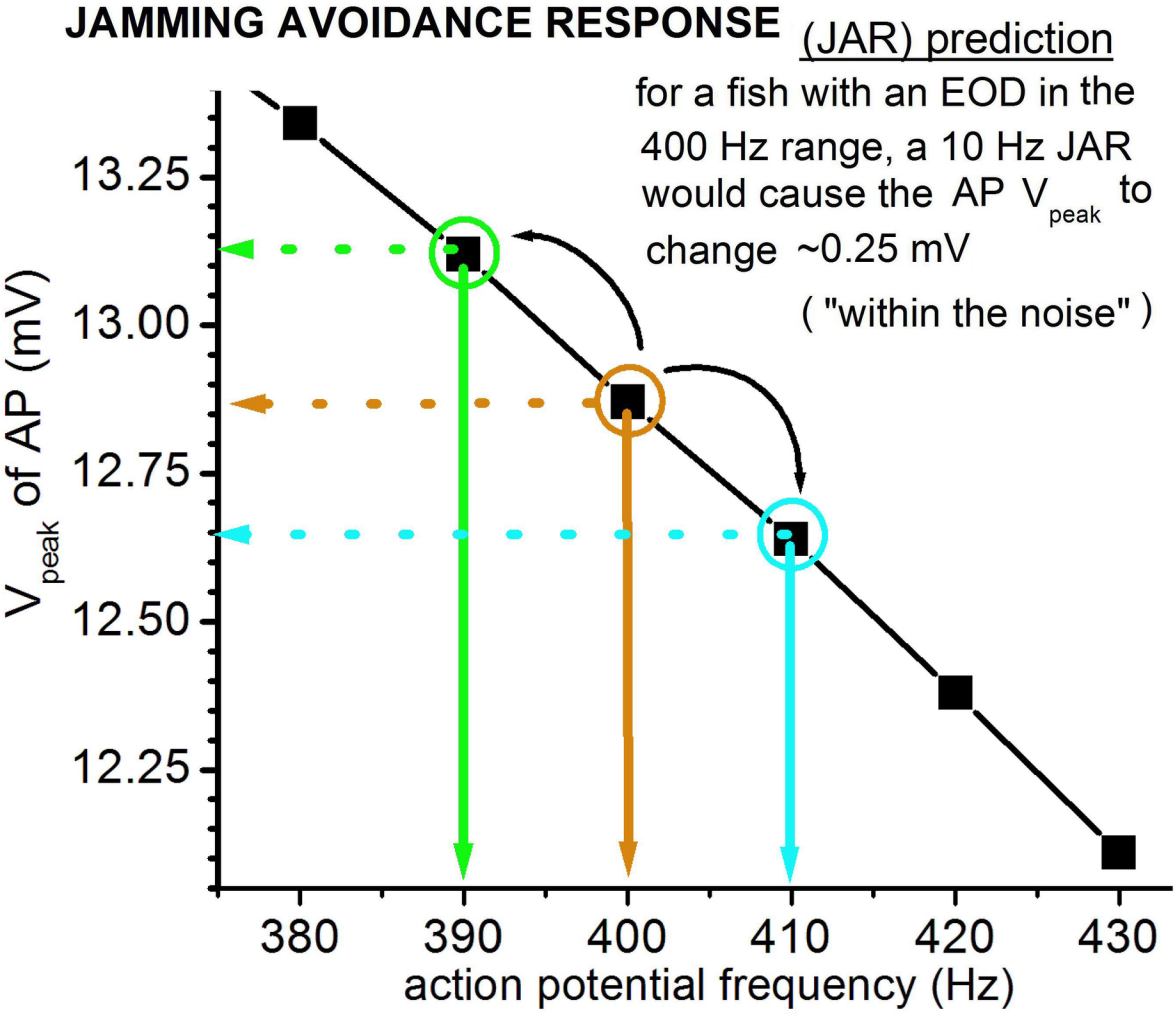
Simulation of a JAR. For APs at frequencies ± 10 Hz near 400 Hz, with gNa_max_ at 783 μS, V_peak_ differs minimally. This suggests that typical ± 10 Hz JARs in *Eigenmannia* might require no increased expression of Nav channels.

In *Eigenmannia* and related electric fish, the close proximity of two fish with similar EOD frequencies produces beat frequencies that are detrimental to electrolocation [37–40]. Fish respond with a jamming avoidance response to increase beat frequency (i.e. increase their frequency differences), wherein the fish with the higher frequency increases its EOD frequency by ~5–10 Hz while the fish with the lower EOD frequency decreases its frequency by a similar magnitude. When fish increase EOD frequency during the JAR, how is the amplitude of the electrocyte AP maintained? To achieve higher AP frequencies for a JAR, *Eigenmannia* individuals could quickly augment the Nav channel density in their electrocytes’ posterior membranes as proposed in earlier work [6]. Based on the Epm however, this costly procedure might be unnecessary. Within this model, extant excitability machinery could produce a typical JAR (i.e. 10 Hz above baseline frequency) with minimal loss of AP integrity. **Fig 8**shows that in a fish with baseline EOD 400 Hz (with gNa_max_ = 783 μS), V_peak_ would change by only ~-0.25 mV during a +10Hz JAR., at 410 Hz, a ΔV_peak_ of ~-0.25 mV. This slightly diminished V_peak_ it should be noted, would be available with no time lag and would reverse immediately when the need for the JAR ended. The difference would be “within the noise” and thus would not be expected to jeopardize EOD signalling integrity.

If JARs occurred frequently, and if signal integrity needed to be even better than the ± 0.25 mV of **Fig 8,** then the issue of JAR dynamic range could be an important determinant of a fish’s “choice” of (central nervous system-determined) stimulus-frequency, given its electrocyte gNa_max._ If a 400 Hz fish operated with a gNa_max_ somewhat greater than 783 μS, then its JAR plot (not shown) would be shallower than in **Fig 8**; likewise across the species frequency range.

### Synaptic stimulation with a non-zero background

On timescales of many hours, *Eigenmannia* individuals rigorously maintain their particular EOD frequency. Thus far (**Figs 5–7**), the background [ACh] in the synaptic cleft (“[ACh]_synaptic_”) is assumed to decay rapidly toward zero after each invariant pulsatile stimulus. But instead, could maintenance of [ACh]_synaptic_ be responsible for setting an invariant frequency? Here we briefly consider this other extreme, plus the more likely situation in which reliably large pulsatile ∆[ACh]_synaptic_ stimuli ride atop a non-zero background [ACh]_synaptic_.

**Fig 9:**
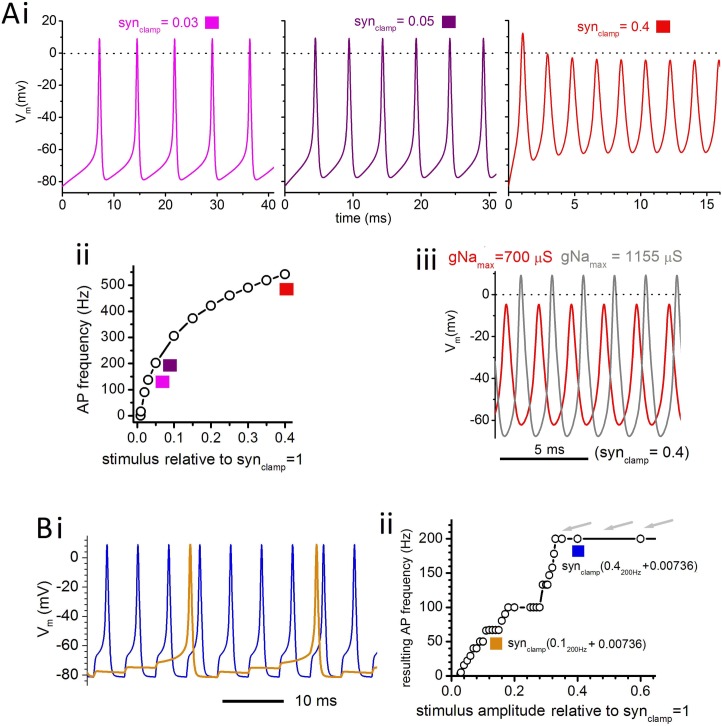
Background synaptic stimulation. **Ai.** Epm APs for three steady-state (i.e. 0 Hz) levels of AChR activation, syn_clamp_0.03_0Hz_, syn_clamp_0.05_0Hz_, and syn_clamp_0.4_0Hz_ fire at 137 Hz, 202 Hz, and 541 Hz respectively (gNa_max_ = 700 μS throughout). **Aii.** Epm AP frequency as a function of stimulus intensity (conditions as in **Ai**); squares color-coded for the plots in **Ai**. **Aiii.** Red trace at 541 Hz firing (conditions as in **Ai**) with a second overlaid trace showing APs when gNa_max_ is increased to 1155 μS (this produces APs of V_peak_ = 9 mV, i.e., the same as for syn_clamp_0.03_0Hz_ with gNa_max_ = 700 μS). **Bi.** APs elicited by a 200 Hz pulsatile stimulus in the presence of a subthreshold background stimulus (syn_clamp_0.00736_0Hz_). For syn_clamp_0.1_200Hz_ (orange) the output AP frequency is 50 Hz whereas for syn_clamp_0.4_200Hz_ (blue) the target frequency of 200 Hz is attained. **Bii.** This background stimulus (syn_clamp_0.00736_0Hz_) plus 200 Hz pulsatile stimuli at the amplitudes indicated elicits APs at different frequencies as shown here in a “devil’s staircase” plot.

**Fig 9Ai** shows Epm APs for various constant or steady-state levels of AChR activation (i.e., 0 Hz stimulation) for gNa_max_ = 700 μS. **Fig 9Aii** shows that a threshold for AP generation occurs at ~syn_clamp_0.0092_0Hz_ (initial point in the plot). As syn_clamp0Hz_ increases above this threshold, the AP frequency rises from 0 Hz until at syn_clamp_0.05_0Hz_ APs (with V_peak_ = 9.0 mV) fire at ~200 Hz, the bottom of the species’ EOD range. With increasing syn_clamp0Hz_ intensity (as with increasing I_clamp_ intensity, see **Figs 5**and **6**) the AP frequency increases but V_peak_ falls (see syn_clamp_0.40_0Hz_ in **Fig 8Ai**) unless, as shown in **Fig 9Aiii**, gNa_max_ is made larger. This shows that, in principle, APs at frequencies required to drive EODs up to 600 Hz could be elicited using a constant-amplitude AChR conductance. In practice, inherent sources of noisy Δ[ACh]_synaptic_ (e.g. as in *Torpedo* electroplax recordings by Giirod et al [24]) would make the EOD frequency unacceptably variable.

In **Fig 9B** APs are stimulated by an imposed Δ[ACh]_synaptic_ that combines pulsatile and background syn_clamp_ (the 0 Hz level was set at 80% of threshold, i.e., syn_clamp_0.0074_0Hz_). **Fig 9Bi** shows V_m_(t) when either syn_clamp_0.1_200Hz_ (orange) or syn_clamp_0.4_200Hz_ (blue) are added atop this 0 Hz background. The former (orange) is insufficient to achieve the target AP frequency (200 Hz) whereas the latter (blue) is sufficient. Over 4 cycles, the syn_clamp_0.1_200Hz_ stimuli summate to trigger 50 Hz APs. When the membrane depolarizes due to Na^+^ entry from pulsed cholinergic stimuli, both transient and persistent gNa changes contribute to maintaining the voltage gain between pulses because in that voltage range inactivation is inconsequential (h≈1). With a fixed subthreshold background (syn_clamp_0.0074_0Hz_) a plot of AP frequency versus 200 Hz stimuli of increasing amplitude generates a “devil’s staircase” plot [41] **Fig 9Bii**). Below the critical pulsatile stimulus amplitude required for any given AP frequency, it would be possible, given a noise-free system, to discern period doubling, tripling etc. In the case illustrated here (i.e., 200 Hz APs) syn_clamp_0.32_200Hz_ is the relevant critical stimulus, and because the modeling is noise-free, period doubling and tripling is evident (plateaus are evident at 100 Hz and 66.6 Hz, i.e. one-half and one-third of the stimulation frequency, 200Hz) in **Fig 9Bii**. For real systems, given the temporal stochasticity of ACh release and the inherent variation in quantal size at the time of release, variations in both pulsatile and background stimulus amplitudes would be expected. This would increase the critical pulsatile stimulus amplitude required for reliable firing at the target frequency; that amplitude would more likely be reached further along the top of the staircase (e.g. at one of the grey arrowheads) than in a noise-free simulation.

### Na^+^ entry for different stimulus regimes

We further wondered how different combinations of background and pulsatile synaptic stimulation would affect Na^+^ entry at frequencies spanning 200 to 600 Hz, the species EOD range (we consider APs of a fixed value, V_peak_ = 13 mV; gNa_max_ was adjusted accordingly). Lewis et al. [6] determined EOD-linked O_2_ consumptions at the whole animal level, not at the level of electrocytes. Here, we stress that from the perspective of whole animal energetics, a Na^+^ ion entering an electrocyte through a Nav channel (gNa) versus a Na^+^ ion entering an electrocyte through an AChR almost certainly presents a different (whole animal level) cost. Given an appropriate Na^+^ gradient, a Nav channel simply has to open in response to depolarization to allow Na^+^ to enter, but for Na^+^ entry via an AChR channel, ACh must first be manufactured and packaged and delivered (at a cost) then released via presynaptic depolarization (at a cost). Accordingly, both total and pathway-specific Na^+^ entry into the electrocyte was tallied. **Table 2**lists outcomes for APs firing at 200 Hz, 500 Hz and 600 Hz. The frequency 500 Hz was included to show how Na^+^ entry/AP evolves as the system approaches its upper limit.

**Table 2:** Na^+^ entry for various synaptic stimulus regimes^∆^.

row #	syn_clamp_ (amplitudes)		gNa_max_^∆^(μS)	Na^+^ entry/AP (x10^9^)		
	**0 Hz part**	**200 Hz part**		**Total entry**	**Nav**	**AChR**
	(thr 0.0092) *			(Nav+AChR)		
**1**	0	1	700	59.2	48.7	10.5
**2**	0.0074	1	698	59.6	48.2	11.4
**3**	0.0074	0.34	835	66.8	61.6	5.2
**4**	0.03	1	714	62.2	48.4	13.8
	(→ 137 Hz)					
**5**	0.03	0.16	824	66.8	60.2	6.6
**6**	0.048*	0	819	67.1	59.6	7.5
	**0 Hz part**	**500 Hzpart**		**Total entry**	**Nav**	**AChR**
	(thr 0.0080)**			(Nav+AChR)		
**7**	0	1	897	70.1	59.8	10.3
**8**	0.0064	1	896	70.2	59.7	10.5
**9**	0.0064	0.68	1015	76.2	68.4	7.8
**10**	0.12	1	925	74.9	59.6	15.3
	(→ 345 Hz)					
**11**	0.12	0.45	1008	78.2	67.1	11.1
**12**	0.28**	0	1097	85.7	71.2	14.5
	**0 Hz part**	**600 Hzpart**		**Total entry**	**Nav**	**AChR**
	(thr 0.0067) ***			(Nav+AChR)		
**13**	0	1	1126	88.3	77.3	11.0
**14**	0.0054	1	1124	88.3	77.1	11.2
**15**	0.0054	0.85	1238	93.8	83.9	9.9
**16**	0.18	1.0	1170	94.4	76.9	17.5
	(→ 414 Hz)					
**17**	0.18	0.7	1219	96.2	80.9	15.3
**18**	0.47***	0	1569	126.0	103.0	23.0

Syn_clamp_ amplitude is non-dimensional (as explained in the section entitled “**Epm responses to pulsatile synaptic current stimulation**”), gNa_max_ is in μS and Na^+^ entry/AP is as defined for **Fig 6B**.

As explained in the text, the 0 Hz background (or subthreshold level) for any set of conditions was chosen to be 80% of threshold for those conditions. Using 75% and 85% of threshold instead yields AP peak amplitudes identical to those for 80% to within 0.01 mV and Na^+^ entry values identical to within 0.2%.

^**∆**^ for each stimulus regime gNa_max_ is adjusted until V_peak_ = 13 mV (hence the threshold values given below vary with frequency)

* syn_clamp_0.0092_0Hz_ brings this system to firing **thr**eshold

syn_clamp_0.048_0Hz_ causes this system to fire at 200 Hz

** syn_clamp_0.0080_0Hz_ brings this system to firing **thr**eshold

syn_clamp_0.28_0Hz_ causes this system to fire at 500 Hz

*** syn_clamp_0.0067_0Hz_ brings this system to firing **thr**eshold

syn_clamp_0.47_0Hz_ causes this system to fire at 600 Hz

The stimulus extremes, i.e. purely pulsatile and purely steady-state (0 Hz) stimulation are represented by rows **1**, **7**, **13** and rows **6**, **12**, **18** respectively. The former reiterates **Fig 6B**, i.e., synaptic components essentially constant across frequencies while Na^+^ entry/AP through Nav channels increases nonlinearly with frequency. For the latter (rows **6**, **12**, **18**), total Na^+^ entry for 0 Hz syn_clamp_ is considerably higher than for pure pulsatile, most markedly so at the high frequency end. Taken together with the argument that 0 Hz syn_clamp_ in vivo would not on its own generate reliable clock-like EODs, it is difficult to conceive of any benefit of a pure 0 Hz regime.

For AP target frequencies 200 Hz, 500 Hz, 600 Hz, the intermediate rows explore situations with some level of [ACh]_synaptic_ persisting between pulsatile stimuli, i.e., combinations of syn_clamp0Hz_ >0 plus a pulsatile syn_clamp_ at some level. For each target frequency, two values of syn_clamp0Hz_ were tested, one subthreshold, the other strongly suprathreshold. Subthreshold was represented by 80% of the 0 Hz threshold (rows **2**, **3**, **8**, **9**, and **14**, **15**), and suprathreshold was represented by the 0 Hz syn_clamp_ amplitude that elicits firing at ~70% of the target frequency (rows **4**, **5**, **10**, **11**, and **15**, **16**). In **Table 2**, footnotes list how, as target frequency increases, the syn_clamp0Hz_ threshold decreases (this occurs because the gNa_max_ imposed to maintain V_peak_ = 13 mV yields a more excitable system).

To provide a basis of comparison in the intermediate (mixed stimuli) rows, each of the two chosen 0 Hz syn_clamp_ amplitudes is first tested in conjunction with the “control” pulsatile amplitude (first column of rows **1**, **7**, **13**) needed for 13 mV AP trains at the target frequency (i.e. rows **2**, **8**, **14**), albeit with gNa_max_ slightly reduced (to maintain V_peak_ = 13 mV). Next (rows **3**, **9**, **15**), with the same subthreshold background, a pulsatile amplitude was found that slightly exceeds the minimum needed to achieve the target AP frequency. For 200 Hz for example, this is slightly rightward of the left margin of the “devil staircase”plateau (**Fig 9Bii**; syn_clamp_0.34_200Hz_ was used here, row **3**). For the “0 Hz suprathreshold-to-70%-target” cases, the Na^+^-entry results are in rows **4** and **5** for 200 Hz, **10** and 11 for 500 Hz, and **16** and **17** for 600 Hz. Thus, across all frequencies, total Na^+^-entry is minimal if there is no steady-state component to the stimulus (rows **1**, **7**, **13**) but if this is not achievable, then the most cost-effective approach appears to be maintaining background synaptic stimulation as low as possible.

The question of which combination of depolarizing Na^+^ entry is most economical will depend on how much more expensive synaptic Na^+^ entry is compared to Nav Na^+^ entry. For example, if Na^+^-entry through AChRs incurred 10X the whole-body metabolic cost of Na^+^ entry through Nav channels, then regimes that reduced entry through AChRs (e.g., rows **3**, **5**, **6** for 200 Hz) would be beneficial. There, the reduced pulsatile component yields a marked saving in pulsatile ACh, though at higher frequency (rows **9**, **15**) the relative benefit diminishes. As **Table 2**shows, the pulsatile syn_clamp_ component required to override the natural firing frequency of the purely fixed amplitude stimulation is larger than for the subthreshold background, and increases with frequency. Nevertheless, at 500 Hz, if the AChR entry pathway was 10X more expensive (as per our suggestion here) then, for rows **10** and **11**, row **11** might be less expensive at the whole-animal level, even though row **10** shows the smaller total Na^+^-entry. In other words, if clearing the synapse of ACh between pulses was not possible, then minimizing the pulsatile amplitude would be beneficial.

Overall, the results of **Table 2**provide some important insights. Between stimulatory pulses (especially at higher frequencies), if electrocyte synapses could not fully remove ACh, an energy efficient system would need to optimize the ratio of background-to-pulsatile amplitude to minimize Na^+^ entry through AChRs. There is no penalty for background ACh provided it is subthreshold. In fact, at low frequency, there is even a saving from a subthreshold background provided the pulsatile component is reduced. Even with a suprathreshold background, the system can fire at reasonable cost, but with costs rising more steeply with frequency than in the case of a purely pulsatile stimulus or with a combined pulsatile and subthreshold background. Even with the caveat that the entry pathway specific differential is as yet unknown, **Table 2**suggests that a moderate background ACh is not detrimental, but that fish generating EODs at the higher end of the frequency range would have relatively little latitude for lowering synaptic costs by increasing the background.

### Calculated electrocyte costs versus whole-animal measurements

Lewis et al. [6] provided whole animal O_2_ consumption data for nine fish generating EODs at frequencies > 300 Hz and < 500 Hz. The data were fit to an exponential. With Epm, we calculated electrocyte Na^+^ entry at frequencies from 200 Hz to 600 Hz. At 200 Hz and below, all Epm parameters return to their pre-stimulus values prior to the next stimulus (in other words, from 0 to 200 Hz, a plot for ATP/AP(Hz) is a flat line. Above that, the values rise non-linearly, though not exponentially. We nevertheless fit an exponential to our Epm-calculated points (for entry through both Nav and AChR channels) at 300, 400 and 500 Hz, and that exponential predicts a 1.17-fold increase between 300 and 500 Hz whereas the Lewis et al. [6] fit over that same range gives a 6.05-fold increase. With Na+ entry/AP through the AChR channels essentially constant across all frequencies (Fig 6), the small but distinct increase with frequency is attributable to influx into the electrocyte through the increasing numbers of Nav channels. However, while Nav channel activation depends only on the voltage of membranes in which the channels are embedded, AChR channel activation depends on a plethora of “upstream” processes whose costs would contribute to whole-animal O2 consumption, but not to the cost of electrocyte ion homeostasis. Therefore, the very large frequency dependent discrepancy (slopes of 6.05 vs 1.17) indicates that the very high cost of EODs at the high end of *Eigenmannia*’s frequency range cannot reasonably be attributed to electrocyte excitability. Instead it suggests that neural events preceding the electrocyte-based APs, along with costs of circulatory processes that help ensure that electrocytes maintain ion homeostasis are substantially responsible. Perhaps this helps explain why the very high frequency *Apteronotid* species have a 'neurogenic' electric organ in which neurally-derived electrocytes receive direct electrotonic inputs without a chemical synapse[42].

## Discussion

Electrocyte action potentials (APs) underlie the wave-type electric organ discharge (EOD) of *Eigenmannia*. This continuous high frequency bioelectrical phenomenon is sustained over the lifespan of the fish. An explanation for how *Eigenmannia* unerringly maintains its EOD requires a better understanding of how the electrocytes produce APs at 200–600 Hz, of the metabolic demands this incurs, and of how subcellular, cellular and tissue geometry along with the associated molecular components are organized to meet these demands [6, 42]. In addition, this knowledge should yield insights for energetic strategies in other highly active excitable tissues including brain and skeletal muscle [17]. Assessing the contribution of electrocyte APs to the whole-animal metabolic cost measured by Lewis et al. [6] requires a thorough accounting of the ionic and biophysical mechanisms of electrocyte APs and post-synaptic currents. Electrophysiology has revealed some of the ionic conductances contributing to electrocyte firing [6, 16], and cyto-histochemistry is showing the locations of ion channels and pumps in the large and highly polarized electrocytes [5]. Here, a computational model for the excitable innervated posterior region of electrocyte membrane is updated to better reflect functional characteristics of electrocytes and to better depict molecular mechanisms of electrocyte excitability.

In the new model, Epm, addresses some limitations inherent in a previous computational model for *Eigenmannia* electrocyte APs. As done previously [16], however, we assumed here that across all EOD frequencies, APs attain the same peak depolarization (V_peak_) and voltage-gated channel kinetics are constant. Again, as previously, the model includes a delayed rectifier conductance (see Methods) even though one has not yet been found in *Eigenmannia* electrocytes. For Epm, we arbitrarily retained the same (frequency-invariant) gK_max_ as previously, then tuned the Hodgkin-Huxley type kinetic parameters for gK(V) and gNa(V) to ensure that sustained APs were possible at 600 Hz, as per the Methods. A major change in the present model of the electrocyte’s innervated posterior membrane is the inclusion of a biophysically appropriate post-synaptic cation conductance. Note that until these specific assumptions are tested, these models are predictive and as a “proof of principle”.

Importantly, adjusting only one parameter in Epm enables the production of APs with consistent amplitude at frequencies up to 600 Hz, the upper end of the species’ EOD frequency range. This parameter is gNa_max_ and corresponds to Nav channel density. Epm exhibits more robust excitability than previous models; it produces all-or-none APs that start from a lower firing threshold, and it fires repetitively with moderate levels of constant current stimulation. It predicts, moreover, contrary to previous models, that electrocytes could achieve a transient jamming avoidance response (this entails a change in discharge frequency of approximately 10 Hz) with no requirement to alter channel properties or channel densities.

The updated model leads to two key predictions and questions for further experimental and computational work. Firstly, a strong prediction from Epm (gleaned from analysis of the distinct Na^+^-entry budgets for Nav and AChR channels and from exploring the costs of varying the densities of both types of channels) is that electrocyte Nav channel density should increase non-linearly with increasing EOD frequency, whereas AChR channel density is expected to be constant with frequency. Secondly, the analysis here indicates that the electrocyte AP itself is a relatively energy-efficient process and is not the major contributor to the whole-animal metabolic cost of EOD production. Instead, the Epm analysis predicts that a much greater proportion of the whole-animal costs associated with EOD production would arise from events “upstream” of the post-synaptic and AP currents. These upstream processes would be those necessary to bring about high frequency activation of the electocytes’ post-synaptic AChRs. Included here would be the continual presynaptic manufacture and vesicular packaging of ACh molecules as well as the costs associated with producing high frequency electromotor neuron and central neuronal pacemaker action potentials [42]. Insofar as the electrocyte’s extensive capillary beds contribute to maintenance of extracellular Na^+^ and K^+^ levels, part of the metabolic costs of continually producing high frequency EOD will also be associated with remote sites such as kidney and gills where circulatory levels of these ions are regulated.

Thus, while Epm applies only to the posterior excitable membrane and assumes that dissipation of E_Na_ occurs only across that membrane, this should not be taken to mean that homeostatic mechanisms that maintain posterior membrane E_Na_ and E_K_ occur exclusively at the posterior membrane. What it implies is that the overall complex (electrocyte/electric organ/circulatory system) is able to regulate the posterior membrane E_Na_ and E_K_ values within very narrow bounds. An important part of the system that has yet to be explored is the posterior and anterior regions’ K-selective inward rectifier channels and the extensive fine t-tubule-like invaginations (given the evolution of electrocytes from skeletal muscle; [32, 43, 44]. Whereas dissipation of electrocyte E_Na_ is a posterior membrane process, dissipation of electrocyte E_K_ is surely an electrocyte-wide process. Ongoing efforts are devoted to developing a full model of the *Eigenmannia* electrocyte that incorporates whole-cell ion homeostasis and the simultaneously occurring processes at both anterior and posterior membranes.

As indicated earlier, Epm includes a necessary “place holder” conductance (the gK(V)) whose physiological role is to achieve fast repolarization of the AP. Ongoing experimental work seeks the molecular identity of this necessary entity. One possibility is an as-yet unidentified K_Na_ channel with rapid voltage dependent kinetics and/or extremely high Na^+^ sensitivity. Another possibility is a fast voltage-gated Cl^-^ channel.

It is self-evident that continual high frequency electrocyte firing with near-zero variance requires near-perfect ion homeostasis and accordingly Epm assumes invariant E_Na_ and E_K_. In principle, the *sine qua non* for perfect ion homeostasis is perfect reciprocal-transport: for each Na^+^ ion that enters, one Na^+^ ion is pumped out. Thus even with no explicit 3Na^+^/2K^+^-ATPase “sodium pump” the model predicts excitability-imposed ATP requirements (and by extension, the required O_2_ consumption) by tallying all excitability-related Na^+^-entry. We again stress, however, that what is not self-evident, is the question of where (i.e., across which membranes and with what geometry) 3Na^+^/2K^+^ pump activity would be best deployed to maintain invariant E_Na_ and E_K_ at the posterior membrane. E_K_ or E_Na_ dysregulation can readily produce pathological patterns of excitability [18] yet astonishingly little is known about the role of cyto-geometry in the Na^+^/K^+^ homeostatic “near-perfection” of high-frequency excitable systems. Epm is therefore a step towards a longer term goal of learning precisely where *Eigenmannia* spends ATP to sustain EODs. Since the 3Na^+^/2K^+^-ATPase is electrogenic, if homeostatic pumping occurred entirely across the same membrane site that generates the high-frequency APs, the dissipative process (ion channel currents) would have to perfectly balance every 3 incoming Na^+^ with 2 outgoing K^+^. Any departure from this pump-imposed constraint would lead to membrane polarization and thus jeopardize the precision of high frequency AP production. If instead, the work of ion homeostasis is partly off-loaded to non-excitable electrocyte regions (e.g., anterior membrane, tubular invaginations of the posterior membrane) and/or to remote organs such as kidney, there would be latitude for diverse flux machinery to offset pump induced polarization (e.g. cation leak channels, inward rectifiers, sodium-activated gK, etc) without jeopardizing the posterior membrane APs. With Epm therefore, we set explicit homeostatic pumping aside to make it possible to quantify (via posterior membrane Na^+^ entry) what the electrocyte achieves as it maintains perfect homeostasis.

Reardon et al [45], examined EOD frequency and amplitude in *Eigenmannia* under reduced water oxygenation. In these experiments hypoxia did not alter EOD frequency, but extreme hypoxia is associated with decreased EOD amplitudes (independent of EOD frequency). This suggests that under these experimental conditions the metabolic cost of EOD signalling may not increase with frequency as dramatically as was observed in later work [6]. This is in line with our present results on AP-related Na+ costs. Note however that Lewis et al measured the direct cost of changing EOD frequency during a jamming responses while EOD amplitude was maintained. So alternatively, EOD frequency could be more vigorously protected (than EOD amplitude) under environmental challenges like hypoxia.

Voltage-clamp data from electrocytes of the lower frequency gymnotid, *Sternopygus*, led to the intriguing suggestion that Nav and Kv channel kinetics are jointly modulated along a continuum such that lower frequency EODs arise from slower channels and higher frequency EODs from faster channels [46, 47]. No confirmatory current clamp data showing AP shapes were obtained, but the idea is to generate at all frequencies, appropriate-shaped APs for yielding sinusoidal EODs. We did not test this as a feature in Epm because, in conjunction with an appropriate pacemaker output, it implies the existence of extraordinarily complex (and perhaps biologically implausible) co-regulation of the kinetics of two discrete channel protein complexes. In any event, use of an invariant set of kinetic parameters in Epm yielded APs whose shape differed little between 200 Hz and 600 Hz. For electrocytes from fish of known EOD frequency, it would be instructive to measure V_m_ while driving the input neuron at different frequencies and to perform current clamp experiments as suggested in **Fig 3.**

## Conclusion

The two core questions driving the current study are broadly applicable to excitable cells in general: 1) what mechanisms enable sustained high-frequency firing? and, 2) what are the relative contributions of membrane excitability, synaptic processes, and organismal-level processes to the metabolic costs of high-frequency firing? The electric organ discharge of *Eigenmannia* is a powerful and accessible system where experimental work on the physiology of EOD production and computational modeling of these physiological processes can form a synergistic and mutually informative approach to answering these questions. An intriguing example of the potentially broad cell physiological relevance of *Eigenmannia* comes to light in the neural processing of high frequency auditory signals [48]. Energetics issues faced by *Eigenmannia* electrocytes arise also for auditory neurons, but to our knowledge how pumps are deployed to ensure post-synaptic homeostasis at high frequency auditory synapses is unknown. Transmission electron microscopy of both *Eigenmannia* electrocytes and auditory synapses reveal abundant but unexplained post-synaptic membrane “vesicles” of unknown function and topology. Perhaps these vesicles are tortuous invaginations that could contribute to ion homeostasis as noted above. For *Eigenmannia*, we showed that this region stains intensely for Na^+^/K^+^ pumps [5] and in skeletal muscle, pumps are an important component of t-tubular membrane. Pursuing the role of these membranous structures in *Eigenmannia* electric organ energetics could, we suspect, shed light on the cellular energetics of high frequency firing in the auditory system.

And looking beyond cell physiology/cell biology, a complete account of the mechanisms and energetics of high frequency firing will have far-reaching implications for understanding processes as varied as the evolution of nervous systems [49, 50], tradeoffs in sensorimotor systems [51] and neuroecological adaptations in animal behavior [52].
